# Screening by high‐throughput sequencing for pathogenic variants in cystic fibrosis: Benefit of introducing personalized therapies

**DOI:** 10.1111/jcmm.17605

**Published:** 2022-11-11

**Authors:** Ana Cristina Vieira de Melo, Karla Simone Costa de Souza, Heglayne Pereira Vital da Silva, Jussara Melo de Cerqueira Maia, Vera Maria Dantas, João Felipe Bezerra, Adriana Augusto de Rezende

**Affiliations:** ^1^ Department of Pediatrics Federal University of Rio Grande do Norte Natal Brazil; ^2^ Department of Clinical and Toxicological Analysis Federal University of Rio Grande do Norte Natal Brazil; ^3^ Technical Health School Federal University of Paraiba Joao Pessoa Brazil

**Keywords:** *CFTR* gene, CFTR modulators therapies, cystic fibrosis, high‐throughput sequencing, variants

## Abstract

This short report documented cystic fibrosis transmembrane conductance regulator (*CFTR*) variants in 37 patients with cystic fibrosis (CF) in the Rio Grande do Norte region of Northeast Brazil. The high‐throughput sequencing technology (HTS) genetic testing provided a definitive molecular diagnosis in 31 patients (83.8%). Among them, 25 patients' carriers of the *c.1521_1523delCTT* variant, categorized as a class 2 mutation, can be currently treated with CFTR modulator drugs. Five children aged 2–5 years could benefit from double lumacaftor/ivacaftor therapy, and 20 patients aged >6 years could be treated with the triple‐combination elexacaftor/tezacaftor/ivacaftor therapy. Thus, the identification of pathogenic variants associated with the development of this disease allows for the introduction of therapy with CFTR modulators that favour better patient management.

## 1 INTRODUCTION

Since the discovery of the association between the *c.1521_1523delCTT* mutation of the cystic fibrosis transmembrane conductance regulator (*CFTR)* gene with cystic fibrosis (CF) development in 1989, technologies for screening *CFTR* gene mutations such as high‐throughput sequencing (HTS), also known as next‐generation sequencing, have assumed a key role in diagnosis and precision medicine based on genotype‐specific small molecule therapies.^1^

This short report describes *CFTR* phenotypes and genotypes post‐HTS application in patients from the Northeast region of Brazil. Furthermore, this report suggests possible personalized therapies that can be adapted according to mutations carried by patients with CF.

## 2 MATERIALS AND METHODS

The study recruited 37 patients (1–37 years) between March 2018 and March 2021 from the CF referral center of Onofre Lopes Hospital University in the Federal University Rio Grande do Norte (UFRN), Natal/RN, Brazil.

The inclusion criteria comprised a CF phenotype, two elevated sweat chloride tests (SCT) (>60 mmol/L) and genomic analysis using a HiSeq 4000 platform. The HTS platform was based on a protocol developed in a multicentre study involving the Brazilian CF Group.^2^

Medical records retrieved for the study included information regarding age, sex, consanguinity, age at onset and diagnosis, body mass index (BMI), phenotypes, SCT values, microbiological analysis of sputum and genetic variants of *CFTR*.

For *CFTR* variants, the Human Genome Variation Society (HGVS) cDNA name was used.^3^, ^4^

The ethics guidelines were established by the research committee of the UFRN and the Declaration of Helsinki (CAAE: 55168816.1.0000.5292). All participants and/or their parents provided signed informed consent.

## 3 RESULTS

Table 1 shows the *CFTR* variants and phenotypes of the 37 patients. A total of 11 pathogenic variants were identified and categorized according to the *CFTR* classification. The most frequent pathogenic variant was *c.1521_1523delCTT*, a processing mutation which results in defective protein folding and trafficking to the cell surface (class 2 mutation). The other variants *c.2988+1G>A*, *c.2989‐3C>G*, *c.2052_2053insA*, *c.1624G>T*, *c.1083_1084insTATGA*, *c.11C>A*, *c.1766G>A*, *c.3266G>A* and *c.3846G>A* belonged to class 1 and encompassed frameshift, nonsense and splicing mutations, resulting in severely reduced or absent *CFTR* expression.^5^, ^6^ Another variant found was *c.3718_2477C>T*, a splice mutation that leads to lower amounts of proteins or a slightly altered proteins that reach the cell surface (class 5 mutation).^5^, ^6^

**TABLE 1:** Phenotypes and *CFTR* variants in 37 patients with cystic fibrosis.

Case	Age (years)	Sex	Age onset (years)	Age diagnosis (years)	Consanguinity	BMI diagnosis	BMI current	Phenotypes	SCT (mmol/L)	PA	Allele 1	Allele 2
1	5	F	0.4	0.6	Yes	Thinness	Adequate	PD/PI/STS/T	92	Yes	*c.1521_1523delCTT*	*c.1521_1523delCTT*
2	4	F	0.5	0.7	Yes	Adequate	Adequate	PD/PI	92	No	*c.1521_1523delCTT*	*c.1521_1523delCTT*
3	8	M	0.1	3	No	Adequate	Adequate	PD/PI	97	Yes	*c.1521_1523delCTT*	*c.1521_1523delCTT*
4	15	F	0.1	2	Yes	Adequate	Adequate	PD/PI/HS	126	Yes	*c.1521_1523delCTT*	*c.1521_1523delCTT*
5	16	F	0.2	5	No	Thinness	Adequate	PD/PI/T/OT	79	Yes	*c.1521_1523delCTT*	*c.1521_1523delCTT*
6	20	M	0.5	0.6	Yes	Adequate	Thinness	PD/PI/RS/T	105	Yes	*c.1521_1523delCTT*	*c.1521_1523delCTT*
7	15	F	0.3	4	No	Thinness	Adequate	PD/PI/RS/BL/RP/T	116	No	*c.1521_1523delCTT*	*c.1521_1523delCTT*
8	21	M	0.1	3	Yes	Thinness	Severe T	PD/PI/RS/HS	61	Yes	*c.1521_1523delCTT*	*c.1521_1523delCTT*
9	35	M	13	13	No	Adequate	Adequate	PD/PI/RS/HS	87	Yes	*c.1521_1523delCTT*	*c.1521_1523delCTT*
10	20	F	2	10	Yes	Severe T	Adequate	PD/PI/RS	68	Yes	*c.1521_1523delCTT*	*c.1521_1523delCTT*
11	14	M	0.1	1.6	No	Adequate	Thinness	PD/PI/RS/HS/T/BL/AE	60	Yes	*c.1521_1523delCTT*	*c.1521_1523delCTT*
12	28	F	0.1	0.5	No	Severe T	Adequate	PD/PI/RS	73	Yes	*c.1521_1523delCTT*	*c.1521_1523delCTT*
13	21	F	1	5	No	Thinness	Thinness	PD/PI/HS/T	67	Yes	*c.1521_1523delCTT*	*c.1521_1523delCTT*
14	28	M	0.6	14	No	Thinness	Adequate	PD/ PI/HS/T/OT	80	Yes	*c.1521_1523delCTT*	*c.1521_1523delCTT*
15	12	F	0.1	0.4	No	Thinness	Adequate	PD/PI/RS/HS/T	72	Yes	*c.1521_1523delCTT*	*c.1521_1523delCTT*
16	21	M	0.1	1.3	No	Severe T	Thinness	PD/PI/RS/MI	63	Yes	*c.1521_1523delCTT*	*c.1521_1523delCTT*
17	11	F	0.1	7.5	No	Adequate	Adequate	PD/PI/RS/BL	58	Yes	*c.1521_1523delCTT*	*c.1521_1523delCTT*
18	4	M	0.1	0.4	No	Adequate	Thinness	PD/PI/T	62	Yes	*c.1521_1523delCTT*	*c.1521_1523delCTT*
19	3	M	0.1	0.3	No	Adequate	Thinness	PD/PI/NC/T	117	Yes	*c.1521_1523delCTT*	*c.1521_1523delCTT*
20	3	F	0.3	0.7	No	Adequate	Adequate	PD/PI	102	Yes	*c.1521_1523delCTT*	*c.1521_1523delCTT*
21	9	M	0.1	6	No	Severe T	Thinness	PD/PI/STS	115	Yes	*c.1521_1523delCTT*	*c.2052_2053insA*
22	21	M	0.1	7	No	Adequate	Adequate	PD/PI	85	Yes	*c.1521_1523delCTT*	*c.11C>A*
23	4	M	0.5	0.5	No	Adequate	Adequate	PD/PI/OT	90	Yes	*c.1521_1523delCTT*	*c.3846G>A*
24	12	M	0.1	1	No	Thinness	Adequate	PD/PI/RS/T/STS/MI/RP	55	Yes	*c.1521_1523delCTT*	*c.3846G>A*
25	15	M	0.1	2	Yes	Adequate	Adequate	PD/PI/RS	115	Yes	*c.1521_1523delCTT*	*c.1766G>A*
26	6	M	0.1	0.3	No	Adequate	Adequate	PD/PI/STS	99	No	*c.1521_1523delCTT*	*c.2989‐3C>G*
27	4	M	0.2	0.5	No	Adequate	Adequate	PD/PI/NC	100	Yes	*c.1521_1523delCTT*	*c.3266G>A*
28	24	F	0.1	0.5	No	Severe T	Adequate	PD/PI/HS/BL	108	Yes	*c.3266G>A*	*c.3266G>A*
29	7	M	0.1	0.2	Yes	Severe T	Adequate	PD/PI/MI	104	Yes	*c.1083_1084insTATGA*	*c.1083_1084insTATGA*
30	4	F	0.1	0.2	No	Adequate	Adequate	PD/PI/AE	109	Yes	*c.1624G>T*	*c.2988+1G>A*
31	25	M	8	22	Yes	Adequate	Severe T	PD/PI	58	Yes	*c.3718_2477C>T*	*c.3718_2477C>T*
32	15	F	5	7	No	Adequate	Adequate	PD/PI/RS	72	Yes	*c.2052_2053insA*	7 T/7 T
33	21	F	0.1	12	No	Adequate	Adequate	PD/RS/STS	67	No	*c.11C>A*	Unknow
34	25	F	0.1	5	No	Thinness	Thinness	PD/PI/T/OT	52	Yes	*c.1521_1523delCTT*	Unknow
35	39	F	14	14	No	Adequate	Adequate	PD/RS	64	Yes	Unknow	Unknow
36	9	M	0.1	4	Yes	Adequate	Adequate	PI/HS/MI	90	No	Unknow	Unknow
37	16	M	9	9	No	Adequate	Adequate	PI	63	No	Unknow	Unknow

*Note*: Body mass index diagnosis classification according the World Health Organization growth charts: Adequate, ≥ − 2 standard deviations (SD) ≤+1 SD; severe thinness <−3 SD; thinness, ≥−3 SD <−2 SD.

Abbreviations: AE, acrodermatitis enteropathy; BL, biliary lithiasis; BMI, body mass index; F, female; HS, hepatic steatosis; M, male; MI, meconium ileus; NC, neonatal cholestasis; OT, otitis; PA, *Pseudomonas aeruginosa*; PD, pulmonary disease; PI, pancreatic insufficiency; RP, rectal prolapse; RS, rhinosinusitis; SCT, sweat chloride test; Severe T, severe thinness; STS, salty‐tasting skin; T, thinness.

Twenty patients were homozygous for *c.1521_1523delCTT* (54.1%) (patients 1–20) and seven were compound heterozygous (18.9%) for *c.2052_2053insA* (patient 21), *c.11C>A* (patient 22), *c.3846G>A* (patients 23 and 24), *c.1766G>A* (patient 25), *c.2989‐3C>G* (patient 26) and *c.3266G>A* (patient 27).

There were four patients (10.8%) without a *c.1521_1523delCTT* mutation: homozygous *c.3266G>A* (patient 28), homozygous *c.1083_1084insTATGA* (patient 29), compound heterozygous *c.1624G>T* and *c.2988+1G>A* (patient 30) and homozygous *c.3718_2477C>T* (patient 31).

In three patients with CF (8.1%), only one pathogenic variant (*c.2052_2053insA*, *c.11C>A*, and *c.1521_1523delCTT*) was detected (patients 32, 33 and 34, respectively). In addition, three patients (8.1%) (patients 35–37) had no variants found by HTS, with CF diagnosis based on their positive SCT and phenotypes.

Based on these genotypes, 25 patients (80.6%) with the *c.1521_1523delCTT* variant were eligible to enhance their respective multidisciplinary therapies with drugs recently approved by the US Food and Drug Administration, which have the potential to correct and potentiate the defective CFTR protein. The patients (aged 2–5 years) with homozygous *c.1521_1523delCTT* were eligible for double‐combination therapy with lumacaftor/ivacaftor (LUM/IVA) (patients 1, 2, 18–20). Twenty patients with CF aged >6 years with two copies of *c.1521_1523delCTT* and/or one copy of *c.1766G>A* could also benefit from both double‐[tezacaftor/ivacaftor (TEZ/IVA)] and triple‐combination therapy [tezacaftor/ivacaftor/elexacaftor (TEZ/IVA/ELX)] (patients 3–17, 21, 22, 24–26).

Patients 23 and 27 could not benefit from triple‐combination therapy because they were <6 years of age. There are yet no modulators that could correct the defects in patients 28–31 with specific variants.

Other patients also had no indication for modulator therapy, as their CF diagnosis was based only on the disease phenotype profile. Patients 32–34, with one copy of a pathogenic variant, were considered to carry CF due to their phenotypes. Finally, patients 35–37, for whom HTS analysis did not reveal any variants, were continued to be monitored by the CF program team.

## 4 DISCUSSION

This short report enrolled 37 patients with CF in the Rio Grande do Norte region of Northeast Brazil. HTS genetic testing provided a definitive molecular diagnosis in 31 patients (83.8%). Among them, 25 patients with the *c.1521_1523delCTT* variant, categorized as a class 2 mutation, and aged >2 years, could be treated with CFTR modulator drugs.

Regarding CFTR modulators, IVA (a first‐generation modulator) is known as a potentiator that binds to defective CFTR on the cell surface, keeping the chloride gate opened; LUM and TEZ (first‐generation modulators) as well as ELX (next‐generation modulator), are known as correctors that bind to different sites on defective CFTR proteins, increasing the number of mature CFTR proteins on the cell surface.^7^, ^8^ Some studies have tested the combination of LUM/IVA and reported rescue of CFTR function with positive results in patients with a homozygous *c.1521_1523delCTT* mutation.

In our study, patients aged 2–5 years (patients 1, 2, 18–20) were eligible for double‐combination LUM/IVA considering their age and high SCT; two of them, patients 18 and 19, had inadequate BMI. In a study by McNamara et al., the use of LUM/IVA in children aged 2–5 years with a homozygous *c.1521_1523delCTT* mutation improved sweat chloride and faecal elastase‐1 concentrations. Additionally, there was an increase in growth parameters and maintenance of adequate scores. However, there was no robust evidence of an improvement in lung function.^9^ Another study by Sagel et al. comprising 169 children (aged > 6 years) and adults demonstrated only improvement in BMI and reduction in SCT, but no difference in pulmonary infections and hospitalizations.^10^

For patients aged >6 years with at least one *c.1521_1523delCTT* copy (patients 3–17, 21, 22, 24–26), the newest triple combination in ELX/TEZ/IVA could be instituted, since the whole group had chronic lung disease, and the therapy would have the potential to reduce hospitalizations. In a trial with 64 children aged 6–11 years, Zemanick et al. reported that treatment improved lung function and BMI and reduced SCT after 24 months.^11^

Patients aged <2 years and carriers of genetic variants that are not targets of CFTR modulator therapies must strictly monitor their nutritional status and lung function and wait for new modulator drugs to be developed that can provide better management of the disease.^12^, ^13^, ^14^

It is noteworthy that the classification of *CFTR* variants plays a key role in personalized therapies. Currently, HTS technology has represented a major advance in CF diagnosis, substituting traditional molecular methods, due to full genotypic screening, enabling the use of precision medicine in the treatment of patients with CF.^15^

## 5 CONCLUSION

This short report documented *CFTR* variants in patients with CF in a region of northeast Brazil, highlighting 25 patients (80.6%) that were eligible for CFTR modulator therapy. Five children aged 2–5 years could benefit from double LUM/IVA, and 20 patients aged >6 years could be treated with the triple‐combination ELX/TEZ/IVA. Thus, HTS technology applied to the identification of pathogenic variants associated with the development of this disease allows for the introduction of therapy with CFTR modulators that favour better patient management. Advanced searches for new modulators are in development and in distinct phases associated with clinical strategies that could benefit every patient with CF.

## AUTHOR CONTRIBUTIONS

**Ana Cristina Vieira de Melo:** Conceptualization (equal); data curation (equal); formal analysis (equal); investigation (equal); methodology (equal); visualization (equal); writing – original draft (equal); writing – review and editing (equal). **Karla Simone Costa de Souza:** Formal analysis (equal); investigation (equal); methodology (equal); visualization (equal); writing – original draft (equal); writing – review and editing (equal). **Heglayne Pereira Vital da Silva:** Formal analysis (supporting); visualization (supporting); writing – review and editing (supporting). **Jussara Melo de Cerqueira Maia:** Formal analysis (supporting); investigation (supporting); visualization (supporting); writing – review and editing (supporting). **Vera Maria Dantas:** Formal analysis (supporting); investigation (supporting); visualization (supporting); writing – review and editing (supporting). **João Felipe Bezerra:** Formal analysis (equal); supervision (equal); visualization (equal); writing – review and editing (equal). **Adriana Augusto de Rezende:** Conceptualization (equal); data curation (equal); formal analysis (equal); funding acquisition (equal); investigation (equal); methodology (equal); project administration (equal); supervision (equal); visualization (equal); writing – original draft (equal); writing – review and editing (equal).

## CONFLICT OF INTEREST

The authors declare that they have no conflicts of interest to disclose.

## Data Availability

All the data supporting underlying findings are included in the manuscript.

## References

[1] Pereira S , Ribeiro J , Ribeiro A , Bertuzzo C , Marson F . Novel, rare and common pathogenic variants in the CFTR gene screened by high‐throughput sequencing technology and predicted by in silico tools. Sci Rep. 2019;9(1):6234. doi:10.1038/s41598-019-42404-6 30996306 PMC6470152

[2] da Silva Filho LVRF , Marostica P , Athanazio R , et al. Brazilian cystic fibrosis patient registry contributors team. extensive CFTR sequencing through NGS in brazilian individuals with cystic fibrosis: unravelling regional discrepancies in the country. J Cyst Fibros. 2021;20(3):473‐484. doi:10.1016/j.jcf.2020.08.007 32819855

[3] Clinical and Functional Translation of CFTR. Accessed May 06, 2022. http://cftr2.org/

[4] Dunnen J , Dalgleish R , Maglott D , et al. HGVS recommendations for the description of sequence variants: 2016 update. Hum Mutat. 2016;7(3):564‐569. doi:10.1002/humu.22981 26931183

[5] Marson F , Bertuzzo C , Ribeiro J . Classification of CFTR mutation classes. Lancet. 2016;4:37‐38. doi:10.1016/S2213-2600(16)30188-6 27377414

[6] Lopes‐Pacheco M . CFTR modulators: shedding light on precision medicine for cystic fibrosis. Front Pharmacol. 2016;7:275. doi:10.3389/fphar.2016.00275 27656143 PMC5011145

[7] Southern KW , Murphy J , Sinha IP , Nevitt SJ . Corrector therapies (with or without potentiators) for people with cystic fibrosis with class II CFTR gene variants (most commonly F508del). Cochrane Database Syst Rev. 2020;12:CD010966. doi:10.1002/14651858.CD010966.pub3 33331662 PMC8094390

[8] Tewkesbury D , Robey R , Barry P . Progress in precision medicine in cystic fibrosis: a focus on CFTR modulator therapy. Breathe (Sheff). 2021;17(4):210112. doi:10.1183/20734735.0112-2021 35035569 PMC8753614

[9] McNamara J , McColley A , Marigowda G , et al. Safety, pharmacokinetics, and pharmacodynamics of lumacaftor and ivacaftor combination therapy in children aged 2–5 years with cystic fibrosis homozygous for F508del‐CFTR: an open‐label phase 3 study. Lancet Respir Med. 2019;7(4):325‐335. doi:10.1016/S2213-2600(18)30460-0 30686767

[10] Sagel S , Khan U , Heltshe S , et al. Clinical effectiveness of Lumacaftor/Ivacaftor in patients with cystic fibrosis homozygous for F508del‐CFTR. A clinical trial. Ann Am Thorac Soc. 2021;18(1):75‐83. doi:10.1513/AnnalsATS.202002-144OC 32644818 PMC7780982

[11] Zemanick E , Taylor‐Cousar J , Davies J , et al. A phase 3 open‐label study of Elexacaftor/Tezacaftor/Ivacaftor in children 6 through 11 years of age with cystic fibrosis and at least one F508del allele. Am J Respir Crit Care Med. 2021;203(12):1522‐1532. doi:10.1164/rccm.202102-0509OC 33734030 PMC8483230

[12] Lee J , Cho A , Huang E , et al. Gene therapy for cystic fibrosis: new tools for precision medicine. J Transl Med. 2021;19:452. doi:10.1186/s12967-021-03099-4 34717671 PMC8556969

[13] Boyd C , Guo G , Huang H , et al. New approaches to genetic therapies for cystic fibrosis. J Cyst Fibros. 2020;19(1):54‐59. doi:10.1016/j.jcf.2019.12.012 31948871

[14] Zaher A , ElSayghJ ED , ElSaygh H , Sanni A . A review of Trikafta: triple cystic fibrosis transmembrane conductance regulator (CFTR) modulator therapy. Cureus. 2021;13(7):e16144. doi:10.7759/cureus.16144 34268058 PMC8266292

[15] Pereira S , Ribeiro J , Ribeiro A , Bertuzzo C , Marson F . Novel, rare and common pathogenic variants in the CFTR gene screened by high‐throughput sequencing technology and predicted by in silico tools. Sci Rep. 2019;9(1):6234. doi:10.1038/s41598-019-42404-6 30996306 PMC6470152

